# Deciphering the mediating role of CXCL10 in hypothyroidism-induced idiopathic pulmonary fibrosis in European ancestry: a Mendelian randomization study

**DOI:** 10.3389/fimmu.2024.1379480

**Published:** 2024-08-09

**Authors:** Xiaoming Xing, Cong Zhao, Song Cai, Jing Wang, Jing Zhang, Fang Sun, Mao Huang, Lishan Zhang

**Affiliations:** ^1^ Dongzhimen Hospital, Beijing University of Chinese Medicine, Beijing, China; ^2^ Department of respiration, Shuguang Hospital, Shanghai University of Traditional Chinese Medicine, Shanghai, China

**Keywords:** hypothyroidism, idiopathic pulmonary fibrosis, Mendelian randomization, CXCL10, immunology

## Abstract

**Background:**

Idiopathic pulmonary fibrosis (IPF) is a lethal lung disease characterized by progressive fibrosis, leading to impaired gas exchange and high mortality. The etiology of IPF is complex, with potential links to autoimmune disorders such as hypothyroidism. This study explores the relationship between hypothyroidism and IPF, focusing on the mediating role of plasma proteins.

**Methods:**

A two-sample Mendelian randomization (MR) approach was employed to determine the impact of hypothyroidism on IPF and the mediating role of 4,907 plasma proteins, all in individuals of European ancestry. Sensitivity analyses, external validation, and reverse causality tests were conducted to ensure the robustness of the findings. Additionally, the function of causal SNPs was evaluated through gene ontology (GO) and Kyoto Encyclopedia of Genes and Genomes (KEGG) pathway enrichment analyses.

**Conclusion:**

The findings suggest that hypothyroidism, through altered plasma protein expression, particularly CXCL10, may contribute to the pathogenesis of IPF. This novel insight highlights the potential of CXCL10 as a therapeutic target in IPF, especially in patients with hypothyroidism. The study emphasizes the need for further research into the complex interplay between autoimmune disorders and IPF, with a view towards developing targeted interventions for IPF management.

## Introduction

Idiopathic pulmonary fibrosis (IPF) is a lethal lung disease characterized by the gradual accumulation of collagen-rich extracellular matrix, culminating in lung tissue fibrosis and the loss of normal alveolar structures (1). This condition leads to progressive, irreversible impairment in gas exchange capacity, ultimately resulting in organ failure and death (2). Affecting approximately 30,000 individuals globally, IPF has a median survival period of merely 3.8 years post-diagnosis (3). The recent advent of antifibrotic drugs, namely pirfenidone and nintedanib, has shown promise in decelerating the disease’s progression (4, 5). Early detection and diagnosis are crucial in substantially enhancing treatment efficacy for IPF patients. However, the precise etiology of IPF remains elusive, with a lack of effective early identification strategies. Emerging studies indicate that IPF’s etiology might involve complex interactions between individual genetic susceptibility and environmental exposures, including smoking, dust, and smoke (6). While not typically classified as an autoimmune disease (7), autoimmune serology positivity is observed in up to a third of IPF patients (8), suggesting a potential role of autoimmune responses in IPF pathogenesis.

Hypothyroidism, a prevalent autoimmune disorder, may be linked to fibrotic diseases. Research by Marks-Garber K et al. revealed that IPF patients are more likely to have been diagnosed with hypothyroidism prior to their IPF diagnosis. Oldham JM et al. discovered a higher prevalence of hypothyroidism among IPF patients compared to those with COPD and the general population, correlating with increased mortality (9). A Mendelian randomization study by Zhang Y et al. established a direct causal relationship between hypothyroidism and IPF onset (10). Nevertheless, the biological mechanisms underlying hypothyroidism’s role in IPF development remain unclear. Thyroid hormones, known to inhibit fibrosis by safeguarding alveolar epithelial cells and restoring mitochondrial function (11), suggest that thyroid hormone supplementation could be pivotal in preventing pulmonary complications in hypothyroid patients (12). However, hypothyroidism’s impact extends beyond diminished thyroid hormone production, influencing oxidative stress (13), autoimmune responses (14), and metabolic dysregulation (15). The complexity of discerning hypothyroidism’s influence on IPF through clinical studies is compounded by the fact that not all hypothyroid patients develop IPF. Plasma biomarkers play a pivotal role in elucidating the mechanisms underlying diseases; however, the levels of these biomarkers can be influenced by thyroid hormone supplementation (16). This interplay of both known and unknown confounders poses significant challenges to the research landscape.

**Figure 1:**
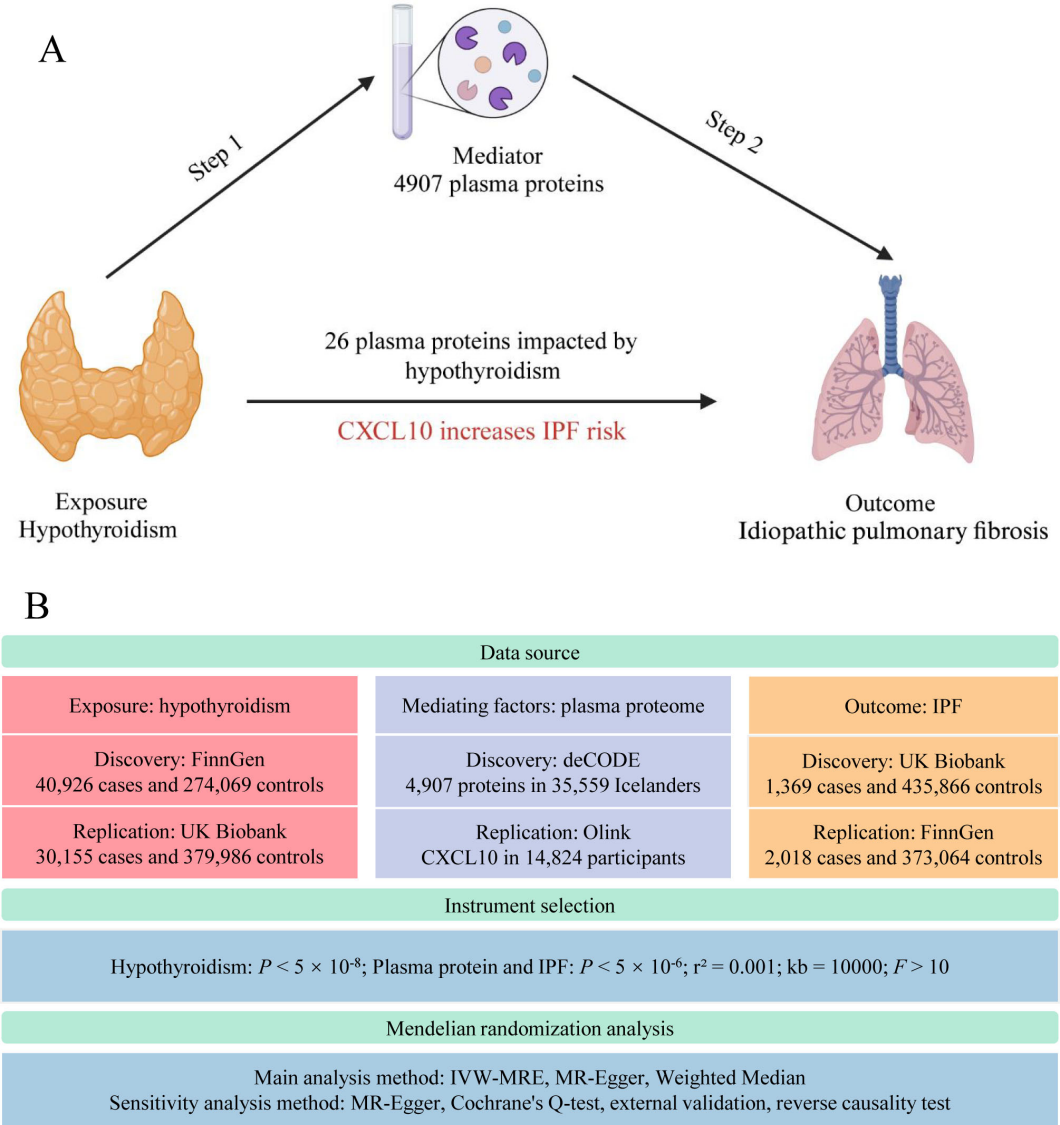
Study flow graph. **(A)** The framework of the two-step MR. **(B)** Overview of data sources and key methodological steps in the study. IPF, idiopathic pulmonary fibrosis; CXCL10, C-X-C motif chemokine ligand 10; IVW-MRE, multiplicative random effects inverse variance weighted. This figure was created by Biorender (https://www.biorender.com/).

Mendelian randomization (MR), employing single nucleotide polymorphisms (SNPs) as instrumental variables, estimates the impact of exposure factors on targeted outcomes. Analogous to the RCT process, SNP generation during meiosis (17) inherently prevents reverse causation and substantially reduces biases inherent in observational studies. As a robust causal inference tool, MR elucidates the interplay mechanisms between diseases. In this study, we conducted a two-step MR analysis using extensive genome-wide association study (GWAS) data on hypothyroidism, pulmonary fibrosis, and the plasma proteome (**Figure 1**). This proteome-wide MR approach identified plasma proteins that mediate hypothyroidism’s influence on pulmonary fibrosis, offering novel insights for clinical interventions.

## Method

### Source of data

The primary analysis of hypothyroidism in this study utilized the GWAS dataset from the FinnGen study R9 (18), where hypothyroidism was defined as “Hypothyroidism, strict autoimmune” (phenocode: “E4_HYTHY_AI_STRICT”), involving 40,926 cases and 274,069 controls of European descent. External validation employed the dataset from Sakaue S et al. of the UK Biobank (19), comprising 3,557 European descent cases and 456,942 controls. Proteomics data were sourced from the Icelandic deCODE study, involving GWAS of 4,907 proteins in 35,559 individuals of European descent (20). The external validation dataset for C-X-C motif chemokine ligand 10 (CXCL10) was sourced from a study by Zhao JH et al., which measured 91 plasma proteins in 14,824 participants using the Olink Target platform (21). The IPF dataset was derived from the UK Biobank data published by Duckworth A et al., including 1,369 European descent cases and 435,866 controls (22). The external validation dataset for IPF was sourced from the FinnGen study R9 (18). Data for free tetraiodothyronine (FT4) and thyroid-stimulating hormone (TSH) were obtained from a GWAS encompassing thyroid characteristics in 22 independent cohort (23). SNPs representing thyroid peroxidase antibodies (TPOAb) were derived from a meta-analysis of TPOAb GWAS involving 18,297 individuals (24).

### Instrument selection

The process of selecting genetic instruments involved several steps. First, we screened for SNPs associated with the exposure factors at genome-wide significance levels (*P* < 5 ×10^-8^). For plasma protein and IPF, the *P* screening threshold was adjusted to 5×10^-6^ due to the scarcity of sufficient SNPs. SNPs exhibiting linkage disequilibrium (r2 = 0.001, kb = 10000, 1000G EUR population) were excluded, and the respective exposure and outcome datasets were aligned using effect allele frequencies. The strength of the genetic instruments was evaluated using *F*-statistics (25). The *F*-statistic is calculated as *F*=(beta/se)^2^, where beta is the estimated effect size and se is the standard error of the SNP-exposure association. To avoid bias in the MR analysis, we excluded weak instrumental variables with *F*-statistics less than 10 (**Supplementary Table 1**).

### MR analysis

#### Step 1: MR analysis of hypothyroidism and plasma proteome

We performed MR analyses to assess the causal effects of the exposure factors on various outcomes. The primary analysis utilized the multiplicative random effects inverse variance weighted (IVW-MRE) method. Additionally, we employed other MR methods, including MR-Egger regression and the weighted median approach, to supplement the IVW estimates and ensure robustness in diverse scenarios. MR-Egger regression was particularly effective in identifying directional pleiotropy, while the weighted median approach yielded consistent estimates, even with some invalid instrumental variables (26). Sensitivity analysis was conducted to evaluate potential pleiotropy and heterogeneity using MR-Egger regression and Cochrane’s Q-test (27, 28).

#### Step 2: assessing the causal relationship between hypothyroidism-affected plasma proteins and IPF

Bonferroni correction was utilized for multiple testing adjustments, setting the significance level at *P* < 0.05/4907. Furthermore, external validation was performed on the proteins identified in the preliminary analysis to confirm the robustness of the results. In the reverse MR analysis, which aimed to evaluate the potential reverse causal relationship between hypothyroidism and the identified protein levels, plasma proteins from the deCODE study were utilized as the exposure variable and hypothyroidism as the outcome, applying a *P*-value threshold of 0.05 for significance. For proteins identified in the initial analysis, two-sample MR analysis was performed. Bonferroni correction was utilized for multiple testing adjustments. Additionally, tests for reverse causation were performed.

### Additional validation of CXCL10

To ensure the robustness of the causal relationship, we performed an additional validation to examine the impact of CXCL10 on hypothyroidism and IPF. This validation aimed to confirm the direction of causality and strengthen the findings of our primary analysis.

### Plasma proteins mediating the link between hypothyroidism and IPF

Considering the complex impact of hypothyroidism on the plasma proteome, our aim was to estimate the proportion of the effect mediated through specific plasma protein levels. Mediation analysis utilizing the product of coefficients method was conducted to determine the extent of mediation by these plasma protein levels in the association between hypothyroidism and IPF. Furthermore, the impact of thyroid hormones on the expression of these proteins was assessed to eliminate potential confounding factors.

### Sample independence

Ensuring sample independence is imperative to avoid weak instrument bias in MR analyses (29). We guaranteed no overlap between subjects in samples used to estimate genetic associations between exposure and outcomes.

### Enrichment analysis of SNP-related genes

Gene names corresponding to each rsID were retrieved from the National Human Genome Research Institute’s Single Nucleotide Polymorphism Database (https://www.ncbi.nlm.nih.gov/snp/). Gene Ontology (GO) and Kyoto Encyclopedia of Genes and Genomes (KEGG) pathway enrichment analyses were conducted using the R packages clusterProfiler (version 4.10.0), org.Hs.eg.db (version 3.18.0), and enrichplot (version 1.22.0). Visualization of the results was facilitated by ggplot2 (version 3.4.4).

### Statistical methods

MR results were presented as effects (beta) or odds ratios (OR) with corresponding 95% confidence intervals (CI), reflecting the impact of each standard deviation increase in the exposure factors on the outcomes. All statistical analyses were conducted using the TwoSampleMR package (version 0.5.7) in R (version 4.3.1). Detailed information on instrumental variable screening and Mendelian randomization methods is available in the **Supplementary Materials**. It is noteworthy that this study exclusively utilized publicly available abstract-level data, eliminating the need for ethical approval.

## Result

### Instrument selection

We selected 131 and 75 SNPs as instrumental variables for two different hypothyroidism datasets, 632 SNPs for 26 proteins (including 21 SNPs for the CXCL10 external validation dataset), 45 SNPs for TSH, 24 SNPs for FT4, 4 SNPs for TPOAB concentration, and 23 SNPs for IPF (**Supplementary Table 1**).

### MR analysis

#### Step 1: outcomes of MR analysis on hypothyroidism and plasma proteome

**Figure 2:**
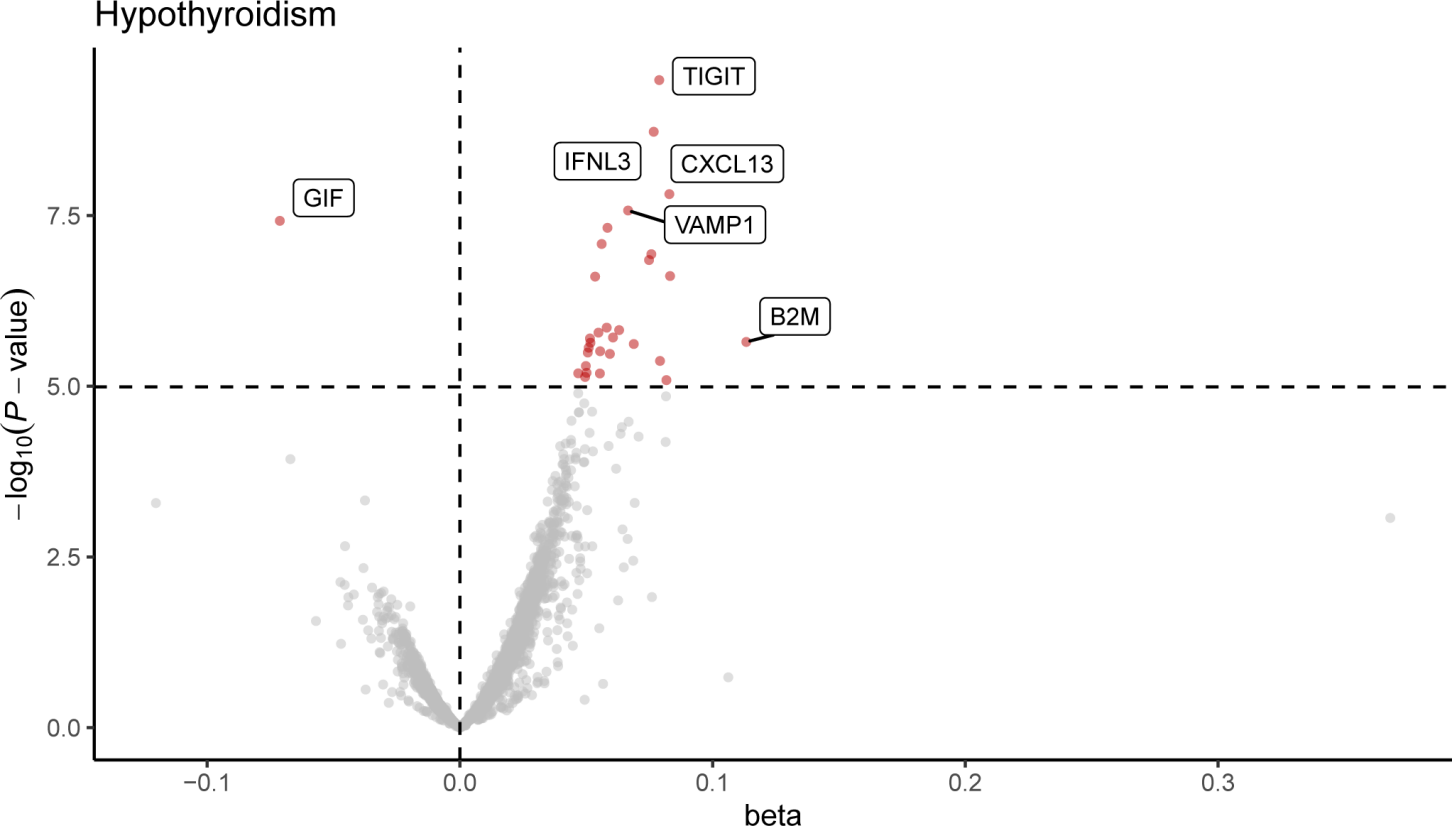
Volcano plot of hypothyroidism-associated plasma proteins identified by MR analysis. This volcano plot depicts the influence of hypothyroidism on the levels of 4,907 plasma proteins, as determined through an inverse variance-weighted Mendelian Randomization (MR) analysis. Each point represents a protein, with the red dots indicating proteins that reached statistical significance at *P* < 1.0 × 10^−5^, following Bonferroni correction for multiple testing (0.05/4,907).

**Figure 3:**
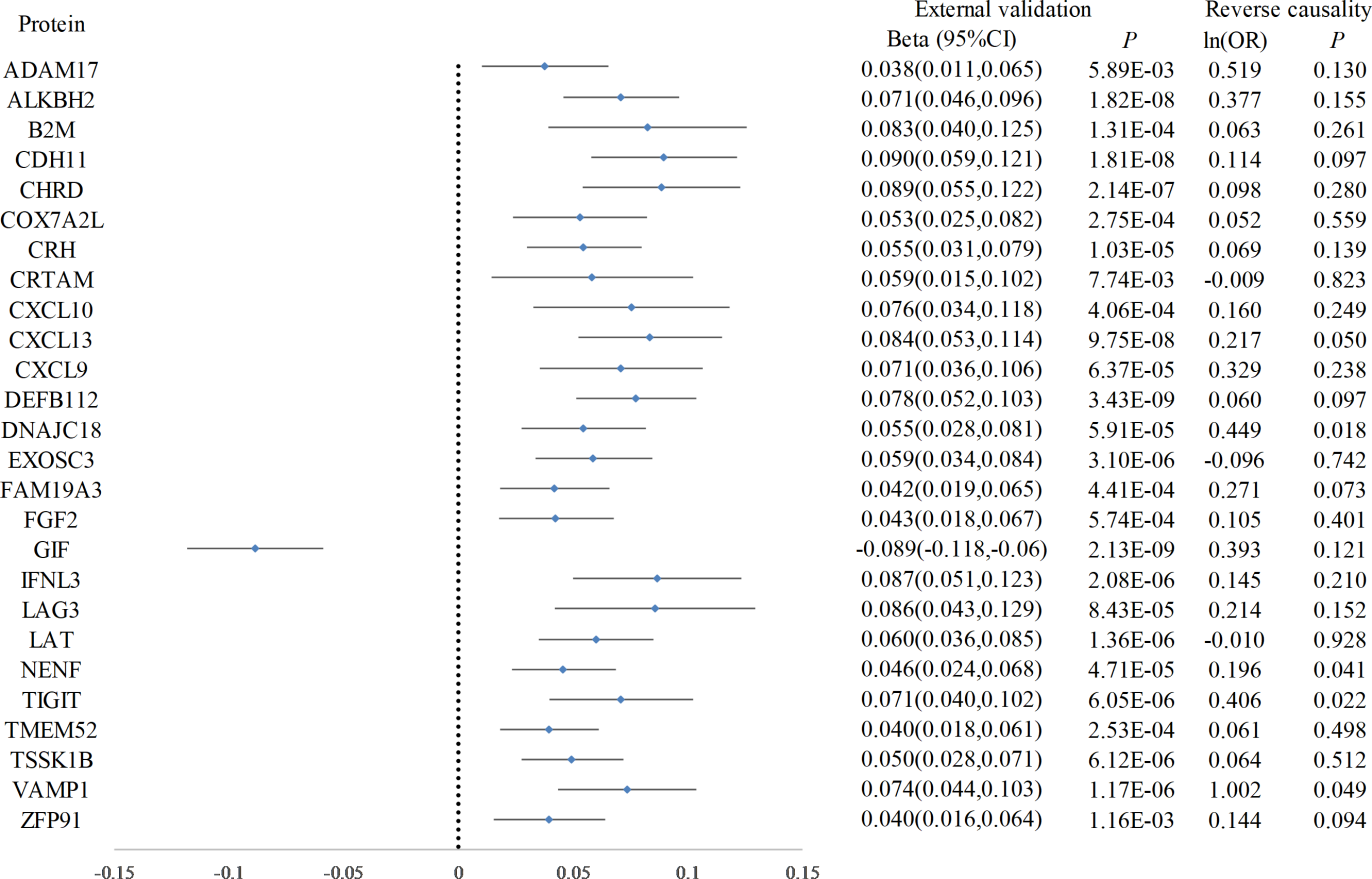
Forest plot of proteins in hypothyroidism with external validation and reverse causation analysis. This forest plot displays the outcomes of a Mendelian Randomization analysis, detailing the influence of elevated hypothyroidism risk on a range of plasma protein levels, as verified in an external validation dataset. Furthermore, annotations on the plot indicate the findings from the reverse causality analysis.

Following Bonferroni correction of the MR results for the plasma proteome using the IVW method, 30 proteins were found to be significantly associated with the risk of hypothyroidism, with an increased risk related to changes in their expression levels (**Figure 2**; **Supplementary Table 2**). Cochrane’s Q-test indicated significant heterogeneity in the instrumental variables for hypothyroidism. MR-Egger regression detected pleiotropy in immunoglobulin superfamily member 11 (IGSF11), adhesion molecule with Ig like domain 1 (AMIGO1), mitochondrial E3 ubiquitin protein ligase 1 (MUL1), and sortilin related VPS10 domain containing receptor 2 (SORCS2), leading to their exclusion from further analysis. External validation verified that 26 proteins, including linker for activation of T cells (LAT), interferon lambda 3 (IFNL3), C-X-C motif chemokine ligand 13 (CXCL13), vesicle associated membrane protein 1 (VAMP1), and CXCL10, met the criteria (**Figure 3**; **Supplementary Table 3**). Additionally, reverse causation assessments were conducted on the proteins identified in the primary analysis. These assessments aimed to determine whether the expression levels of these proteins could influence the occurrence of hypothyroidism. However, our analysis revealed significant reverse causal relationships for CXCL9, ADAM metallopeptidase domain 17 (ADAM17), T cell immunoreceptor with Ig and ITIM domains (TIGIT) and beta-2-microglobulin (B2M), indicating a more complex interplay than previously understood (**Supplementary Table 4**). The lack of reverse causation supports the likelihood of a unidirectional relationship, suggesting that the pathogenesis of hypothyroidism may influence changes in protein expression, rather than being a reverse outcome.

#### Step 2: causal effects of hypothyroidism-influenced plasma proteins on IPF

Subsequently, we reassessed the causal impact of the identified hypothyroidism-driven proteins on IPF outcomes using two-sample MR. Qualified protein quantitative trait locis (pQTLs) of these proteins were utilized as instrumental variables, and pQTLs of 26 hypothyroidism-driven proteins were identified using the deCODE study, testing their causal effect on pulmonary fibrosis in MR. In our MR analysis, a notable association was observed between elevated CXCL10 levels and an increased risk of IPF, although this did not meet the stringent Bonferroni-adjusted threshold (ln(OR) = 1.62×10^-3^, *P* = 4.25×10^-3^). Other proteins analyzed did not demonstrate associations with IPF risk at the conventional significance level of *P* < 0.05 (**Table 1**; **Supplementary Table 5**). Additionally, our analysis did not establish a significant reverse causal relationship between IPF and CXCL10 (beta = 5.694, *P* = 0.070), indicating the need for further studies to explore these potential links (**Supplementary Table 6**).

**Table 1:** Causal Links between Hypothyroidism-Influenced Proteins and IPF.

Protein	Primary analysis		Reverse causality	
	ln(OR)	*P*	Beta	*P*
ADAM17	-1.50E-05	0.990	-0.199	0.949
ALKBH2	4.90E-04	0.550	0.026	0.992
B2M	7.57E-04	0.175	0.542	0.842
CDH11	1.06E-04	0.671	-2.934	0.305
CHRD	1.00E-03	0.085	2.027	0.565
COX7A2L	2.48E-04	0.588	-3.703	0.213
CRH	1.23E-03	0.050	-0.278	0.936
CRTAM	1.50E-05	0.969	0.621	0.869
CXCL10	1.62E-03	0.004	5.694	0.070
CXCL13	3.09E-04	0.670	1.827	0.475
CXCL9	1.67E-04	0.778	3.712	0.230
DEFB112	2.41E-04	0.603	-1.536	0.516
DNAJC18	2.63E-04	0.738	1.243	0.609
EXOSC3	3.92E-04	0.330	1.451	0.644
FAM19A3	4.96E-04	0.616	6.435	0.006
FGF2	1.65E-04	0.507	-1.831	0.465
GIF	1.45E-04	0.649	4.894	0.036
IFNL3	2.26E-04	0.758	4.672	0.152
LAG3	1.24E-04	0.731	1.543	0.622
LAT	5.61E-04	0.416	9.358	0.004
NENF	9.29E-04	0.244	2.191	0.422
TIGIT	1.66E-04	0.828	0.425	0.889
TMEM52	-1.38E-03	0.056	-0.550	0.810
TSSK1B	6.26E-04	0.424	1.627	0.475
VAMP1	6.64E-04	0.402	6.502	0.007
ZFP91	2.61E-04	0.835	3.073	0.181

### Additional validation results for CXCL10

We conducted an independent analysis using an external dataset for CXCL10 to evaluate its impact on hypothyroidism and IPF. Our findings indicated that elevated expression of CXCL10 was associated with an increased risk of IPF (ln(OR) = 0.29, *P* = 0.03), whereas no significant effect was observed on hypothyroidism (ln(OR) = 0.25, *P* = 0.14). Additionally, we analyzed the effect of IPF on CXCL10 expression and found no significant impact (ln(OR) = 0.27, *P* = 0.95). These results are consistent with those obtained in our primary analysis, thereby reinforcing the validity of our conclusions.

### CXCL10 mediating the link between hypothyroidism and IPF

**Figure 4:**
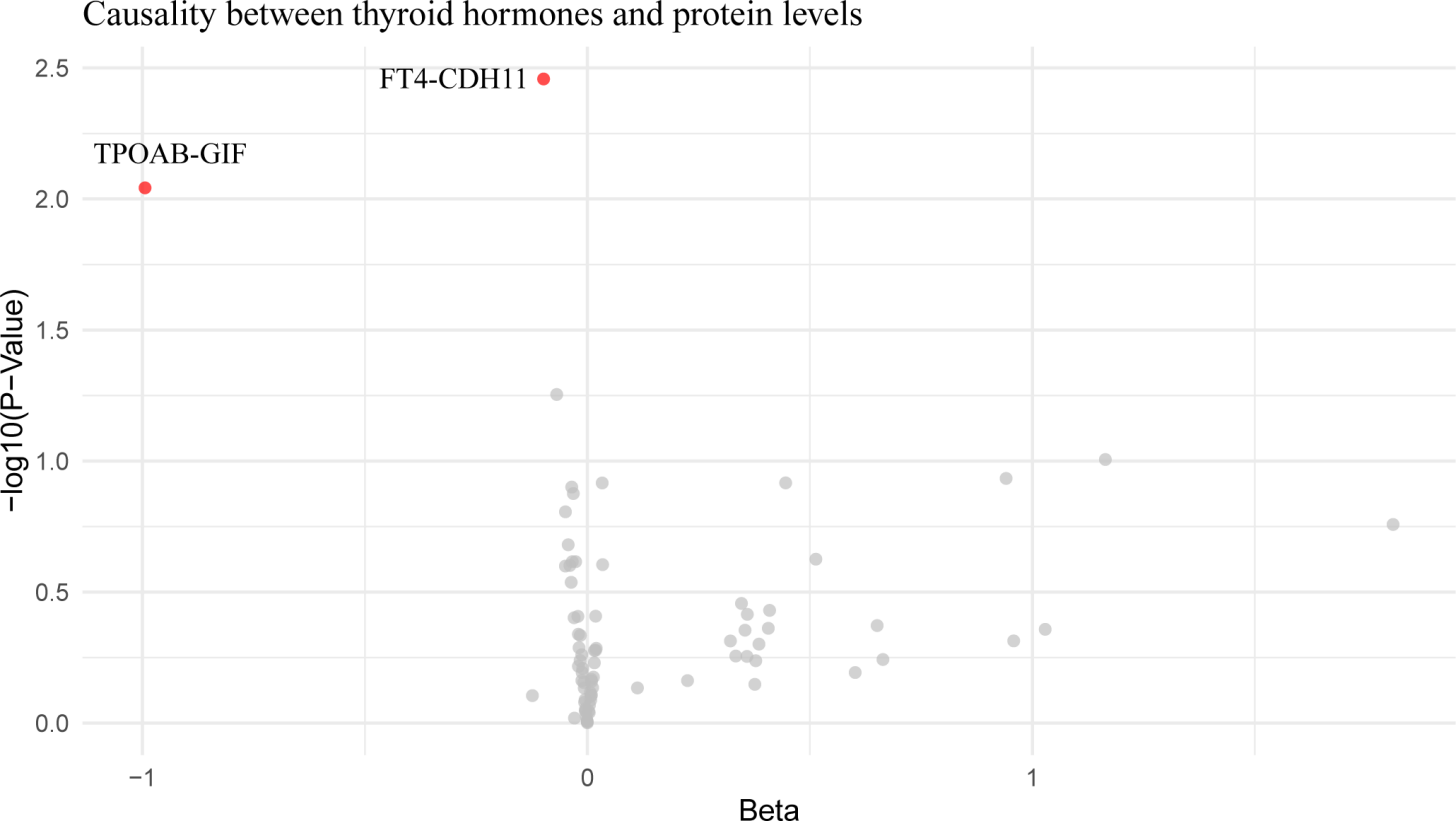
Volcano plot of thyroid hormone effects on hypothyroidism-influenced proteins. This volcano plot graphically represents the impact of thyroid hormone levels on 26 plasma proteins that are influenced by hypothyroidism, as determined through an inverse variance-weighted Mendelian Randomization analysis. Each dot on the plot symbolizes a distinct protein, with the red dots highlighting those proteins that demonstrate statistically significant changes (*P* < 0.05).

Mediation analysis revealed that CXCL10 accounted for 36.45% of the impact of hypothyroidism on IPF. Additionally, an increase in FT4 was found to reduce Cadherin 11 (CDH11) expression, and an increase in TPOAB concentration to reduce gastric intrinsic factor (GIF) expression (**Figure 4**; **Supplementary Table 7**), but these proteins were not found to affect pulmonary fibrosis in subsequent analyses.

### Functional evaluation of causal SNPs

To further elucidate the functional roles of the instrumental variable SNPs for hypothyroidism and CXCL10, we analyzed the candidate genes corresponding to each rsID (**Supplementary Table 8**). Comprehensive insights into the biological processes associated with these genes were provided through GO enrichment analysis and KEGG pathway analysis.

**Figure 5:**
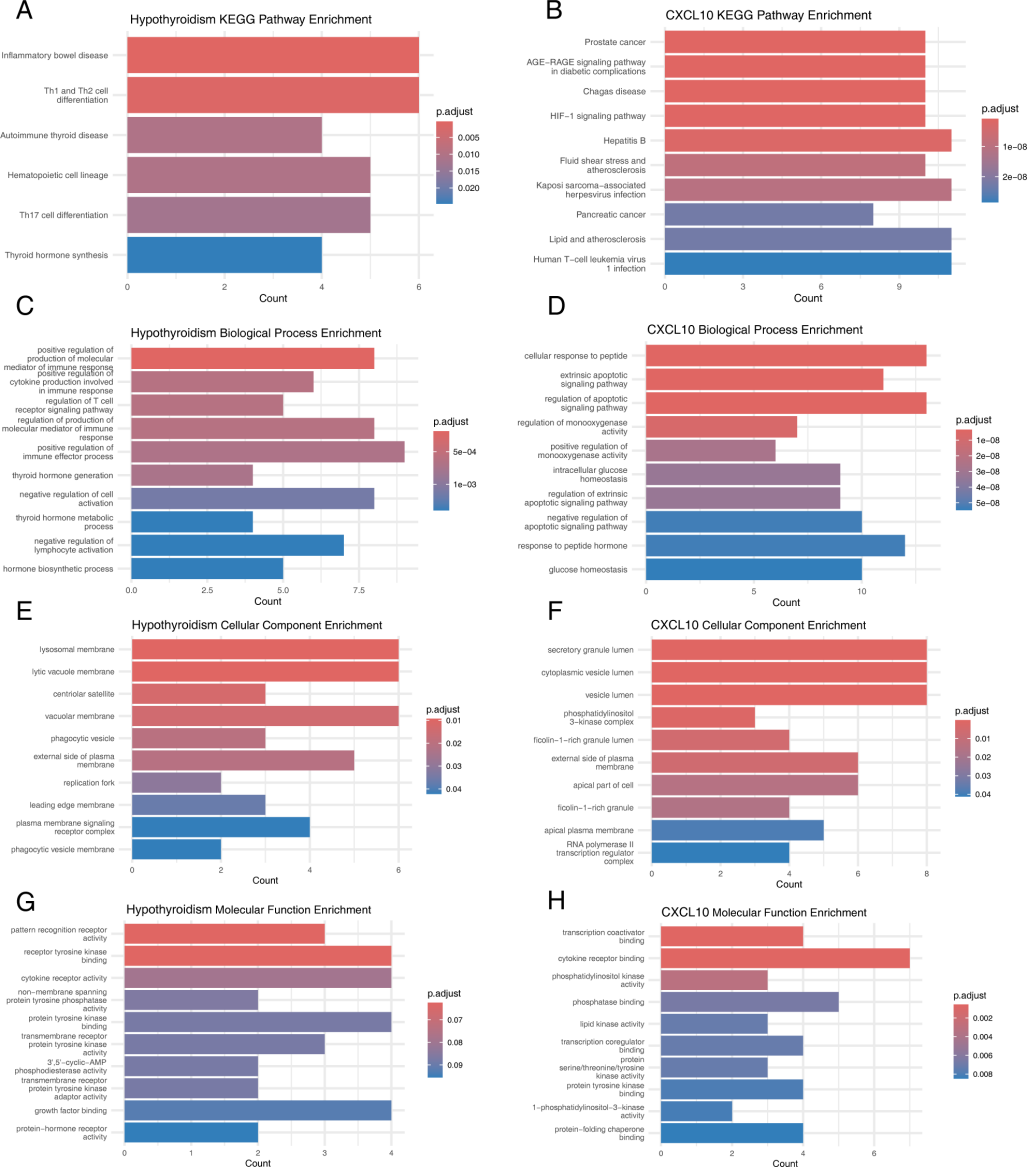
Functional evaluation of causal SNPs. **(A)** KEGG Pathways for Hypothyroidism-Related Genes. **(B)** KEGG Pathways for CXCL10-Related Genes. **(C)** GO Biological Processes for Hypothyroidism-Related Genes. **(D)** GO Biological Processes for CXCL10-Related Genes. **(E)** GO Cellular Components for Hypothyroidism-Related Genes. **(F)** GO Cellular Components for CXCL10-Related Genes. **(G)** GO Molecular Functions for Hypothyroidism-Related Genes. **(H)** GO Molecular Functions for CXCL10-Related Genes.

KEGG pathway analysis revealed that candidate genes for hypothyroidism were predominantly enriched in pathways such as inflammatory bowel disease, Th1 and Th2 cell differentiation, autoimmune thyroid disease, hematopoietic cell lineage, Th17 cell differentiation, and thyroid hormone synthesis (**Figure 5A**). In contrast, CXCL10 candidate genes were mainly enriched in pathways including prostate cancer, AGE-RAGE signaling pathway in diabetic complications, Chagas disease, HIF-1 signaling pathway, hepatitis B, fluid shear stress and atherosclerosis, Kaposi sarcoma-associated herpesvirus infection, pancreatic cancer, lipid and atherosclerosis, and human T-cell leukemia virus type 1 infection (**Figure 5B**).

GO enrichment analysis showed that hypothyroidism candidate genes were significantly enriched in biological processes such as positive regulation of the production of molecular mediators of immune response, positive regulation of cytokine production involved in immune response, regulation of T cell receptor signaling pathway, thyroid hormone generation, endocytic vesicle membrane, lamellipodium, WASH complex, pattern recognition receptor activity, receptor tyrosine kinase binding, and cytokine receptor activity (**Figures 5C, E, G**). Similarly, CXCL10 candidate genes were enriched in cellular response to peptide, extrinsic apoptotic signaling pathway, regulation of apoptotic signaling pathway, secretory granule lumen, cytoplasmic vesicle lumen, vesicle lumen, transcription coactivator binding, cytokine receptor binding, and phosphatidylinositol kinase activity (**Figures 5D, F, H**).

## Discussion

Prior observational studies have demonstrated a significant association between hypothyroidism and an increased likelihood of liver, heart, and lung fibrosis diseases (9, 30). However, owing to the influence of thyroid hormones on the development of pulmonary fibrosis (31, 32), the causal relationship between these two diseases continues to be controversial. Zhang Y et al. directly demonstrated a causal relationship between the genetic susceptibility to hypothyroidism and the occurrence of IPF (10). However, it is still unclear how hypothyroidism affects the development of IPF, apart from its impact on thyroid hormone release. In this study, utilizing MR analysis for the first time, we discovered that hypothyroidism can lead to changes in the expression levels of 26 plasma proteins, including CXCL13, VAMP1, and CXCL10, among which CXCL10 may mediate the impact of hypothyroidism on the risk of developing IPF. The activity of the thyroid and thyroid autoantibodies had no impact on the expression of these proteins, indicating that CXCL10 may be a key mediator protein in the impact of hypothyroidism on IPF.

In the United States, the rates of overt and subclinical hypothyroidism are 0.4% and 9%, respectively, while in women over 75, the rate of subclinical hypothyroidism can exceed 20% (33). Its mechanism entails T-cell-mediated organ-specific autoimmune responses (34), characterized by lymphocyte infiltration into thyroid tissue and direct alteration of thyroid follicular cell function through the action of Interleukin 1 (IL-1), tumor necrosis factor (TNF), and interferon γ (IFNγ) (35). The secretion of chemokines induced by IFNγ is also associated with lymphocyte infiltration in the thyroid (36). The results of this study align with previous clinical research, demonstrating that genetic susceptibility to hypothyroidism leads to changes in the expression levels of various proteins, including chemokines such as CXCL10 (37) and CXCL13 (38), previously shown to be elevated in the plasma or thyroid tissue of hypothyroidism patients. Our study reemphasizes that autoimmune diseases can affect protein expression throughout the body through abnormal immune responses, leading to pathologies in other sites. KEGG pathway analysis and GO enrichment analysis revealed that hypothyroidism and CXCL10-related genes intersect at cytokine receptors and the endocytic vesicle membrane. Other researchers have also identified a dynamic interaction between thyroid hormones and the immune response, which can mediate different autophagic targets, leading to the development of various diseases (39, 40).

CXCL10, a chemokine that binds to its specific receptor C-X-C motif chemokine receptor 3 (CXCR3), mobilizes and activates immune cells such as T cells, monocytes, and NK cells in specific tissues, playing a crucial role in tissue damage in autoimmune diseases (41). The interplay between CXCL10 and CXCR3 is crucial for the Th1 immune response. Thyroid follicular cells, stimulated by inflammatory factors such as IFN-γ and TNF-α, produce CXCL10, inducing Th1 lymphocyte migration to thyroid tissue and further production of inflammatory factors (42). This cycle is involved in the development of autoimmune thyroiditis (43) and leads to subsequent thyroid destruction and hypothyroidism (44). The CXCL10–CXCR3 axis has been shown to regulate the activation of pulmonary fibroblasts, affecting extracellular matrix production (45). CXCL10 can cause an imbalance between anti-fibrotic Th1 cells and pro-fibrotic Th2 cells (46), affecting tissue repair mechanisms and fibrotic remodeling, leading to the development and progression of IPF (47, 48).

Remo C. Russo and colleagues proposed that the activation of alveolar macrophages might generate inflammatory and fibrotic chemokines (CCL8, CCL13, CXCL10, etc.) in the pulmonary microenvironment. These chemokines can regulate angiogenesis, induce chronic inflammation, and promote leukocyte influx and activation, as well as fibroblast recruitment and myofibroblast activation, leading to fibrosis (49) and participating in tissue repair and scar formation (50). Additionally, lymphocyte expression of CXCL10 leads to leukocyte influx, causing lung tissue damage and scar formation (51). However, this does not fully explain the source tissue of CXCL10. Our study revealed that susceptibility to hypothyroidism elevates plasma CXCL10 expression, leading us to speculate that chemokines may act as a bridge linking thyroid dysfunction with pulmonary tissue remodeling and fibrosis in IPF. In SLE, Nielepkowicz-Goździńska A and others found that CXCL10 and CXCL11 are associated with the accumulation of neutrophils in the alveolar spaces of patients with SLE lung fibrosis, potentially contributing to interstitial fibrosis (52), providing some support for our hypothesis.

This study employed a two-step MR approach, using large-scale plasma proteomics as an intermediary factor to explain the impact mechanism of hypothyroidism on IPF. We identified that genetic susceptibility to hypothyroidism raises plasma CXCL10 expression levels, and CXCL10, in turn, increases the risk of IPF. Therefore, we speculate that CXCL10 could be a potential intervention target for preventing IPF in patients with hypothyroidism and emphasize that chemokines may be intermediary factors in how autoimmune diseases affect other systemic diseases. This study further strengthened the robustness of MR results through external validation, reverse testing, and multiple sensitivity analyses. However, this study has several limitations. Although thyroid hormones may influence the occurrence of IPF, our research primarily focused on the autoimmune characteristics of hypothyroidism. Previous studies have shown that PPAR-α activators can suppress the secretion of CXCL10 and CCL2 in thyroid cells (53). Future research should investigate whether the use of PPAR-α activators in hypothyroidism patients impacts the subsequent development of IPF. Additionally, there is currently a lack of prospective studies examining the risk of IPF in patients with hypothyroidism. To address this gap, we plan to utilize the UK Biobank cohort to further explore the relationship between hypothyroidism, CXCL10, and IPF. Moreover, our study was limited to individuals of European descent due to the lack of protein GWAS data in non-European populations. Expanding research to include diverse populations will be crucial for understanding the broader implications of our findings and ensuring the generalizability of the results.

In summary, our research presents compelling evidence that CXCL10 is a critical mediator in the nexus between hypothyroidism and IPF. This finding not only enhances our understanding of the pathophysiological mechanisms underlying these conditions but also positions CXCL10 as a promising therapeutic target in the treatment of IPF. The potential of CXCL10 modulation as a novel intervention strategy offers an exciting avenue for future drug development, underscoring the need for further experimental studies to substantiate our results and explore this therapeutic potential. Moreover, broadening the scope of our investigation to encompass a wider spectrum of autoimmune disorders and fibrotic diseases could yield invaluable insights. Such research would deepen our comprehension of the overarching role that chemokines play in mediating these complex conditions, potentially unlocking new pathways for intervention and treatment.

## Data Availability

The original contributions presented in the study are included in the article/**Supplementary Material**. Further inquiries can be directed to the corresponding authors.
